# Hospital and Institutional News

**Published:** 1920-06-19

**Authors:** 


### HOSPITAL AND INSTITUTIONAL NEWS.

### Sir Napier Burnett on the Hospital Crisis.

### TRUE SITUATION NOT SHOWN BY FIGURES.

FURTHER reference to the progress of his survey of provincial hospitals, undertaken for the British Red Cross and Order of St. John, is made by Sir Napier Burnett in an article in *The Times* of Wednesday last. The latest survey figures refer to the expenditure and income of 543 of the 640 hospitals of the provinces. It is shown that the excess of ordinary expenditure over ordinary income in the institutions listed (which no doubt include the most important ones) during 1919 was £475,627, or fixing a likely approximate figure for the whole of the provincial hospitals, £600,000 for the year. But a point which it is necessary to emphasise is brought out very clearly, that the money deficit is not the true deficit. Many of the hospitals admit that in view of the extra cost of labour and materials they have refrained from carrying out the necessary repairs, alterations, and improvements that have fallen into arrears since the beginning of the war. Further, many of the hospitals have been " carrying on " with plant much of which is out of date. For instance, in some cases new laboratory provision is needed, new *x*-ray plant, fresh operating theatre, appliances, etc., all necessary to bring the institutions up to the standard of modern requirements and to permit of the patients obtaining the maximum benefit from the treatment employed.

### ECONOMY OF BEDS.

Sir Napier Burnett numbers amongst his most important points economy in the use of beds, and he draws attention once more to the curious anomaly that, while there is a large waiting-list nearly everywhere, there is a constantly large percentage of unoccupied beds. The pressure, it is pointed out, is undoubtedly greater in some localities than in others, and Sir Napier suggests that it is sufficiently widespread and acute as to justify the need for an official inquiry on a broader and more detailed basis than that of the present survey. Such an inquiry should be undertaken by some Commission visiting the more important areas, and ascertaining on the spot the actual requirements, and how best they could be met. This should be undertaken before the hospitals launch the new building schemes they now have in contemplation, and also before the Government finally decides on its hospital policy. More systematic use of the 12,000 convalescent beds is advocated in order that the patients may be discharged as soon as their chief medical needs have been supplied, and may then proceed to their own homes *via* the convalescent hospital.

### SCARCITY OF NURSES.

Complaints as to shortage of nurses in both the large and small hospitals are widespread, and in even the greater hospitals any choice of probationers is almost impossible. The greater number of hospitals indicate not only a deficient supply, but also that the general standard of the applicants is not of a high order. The following reasons in explanation of the present falling off in the supplyof hospital nurses are suggested. The question of salary: example, a trained masseuse, in about a third of the time it takes to qualify as a nurse, can command double a nurse's salary. Health visiting and school medical services, etc., are absorbing women who would have been eligible to work as nurses. Long hours of ward work. Social welfare and general development of the nurse's life are neglected by hospital authorities. Sir Napier suggests that the Ministry of Health or Board of Education might supply money grants to hospitals for the training of nurses, as is now done in the case of lying-in institutions. Such grants might be apportioned in part to the hospital and in part supplement the nurse's salary. In this way it would be possible for the central authority to exercise some control over the general standard of training, which varies greatly in different institutions.

### HIS MAJESTY AT MILLBANK.

LAST week the King visited Millbank Hospital, in order to inspect memorial panels and a recreation room, provided out of funds generously given by Major W. N. Keeper, of Toronto, late of the Indian Medical Service, to be expended at his Majesty's discretion for the relief of suffering caused by the war. The King took the opportunity of conferring a decoration upon a notable representative of the American Army Medical Service, General Gorgas, who has been made a Knight Commander of St. Michael and St. George. On his way through the general wards the King visited Rough Rider Lucking, from the Royal Mews at Windsor, who was taken to the hospital from the Royal Tournament at Olympia, and the sailor whose hand was injured in one of the competitions at the Tournament. The panels provided by Major Keeper illustrate the work of the R.A.M.C. The first shows the stretcher-bearers bringing in wounded from the front line, the second treatment in the advanced dressing-stations, the third the patient being conveyed from the dressing-station, and the fourth is a picture of a hospital train.

### THE DUKE OF CONNAUGHT.

A DEPUTATION from the Royal College of Surgeons in Ireland, consisting of Mr. Edward H. Taylor (President), Sir William de Courcy Wheeler, F.R.C.S. (Vice-President), Mr. John B. Storey, F.R.C.S. (ex-President), and Mr. Alfred Miller (Registrar), conferred on June 11, at Clarence House, on the Duke of Connaught the Diploma of the Honorary Fellowship of the College, to which His Royal Highness was last year elected.

### A PLAN FOR CONCERTED ACTION.

WE hope that the various schemes now on foot in London to raise the necessary income for the voluntary hospitals will not get in one another's way.. There is the Hospital Saturday Fund, with the programme to which we lately alluded. Then the committee formed by the British Hospitals Association has not yet reported. Then there is the Red Cross Appeal, with its foreshadowing of concerted action later. Nothing much will be done until the British Hospitals Association has produced its committee's report, but much time will have been saved if this report shall include the plan for concerted action to which so many tentative efforts are being made. We know sufficiently the present income and expenses of the London hospitals. We know that an extra million a year is required to balance their accounts. That which remains to be decided is the precise part which King Edward's Hospital Fund, the League of Mercy, the Hospital Saturday Fund, the Hospital Sunday Fund, shall severally and jointly take, *in addition to* their usual activities, to raise it. The Red Cross, with its wider net, has already started.

### A CHAIR IN BIO-CHEMISTRY.

It is officially announced at Oxford, upon the nomination of Mr. Edward Whitley, who gave £10,000 towards the endowment of the Chair of Bio-chemistry, with the approval of the Vice-Chancellor, the Regius Professor of Medicine, the Waynflete Professor of Chemistry and Physiology, etc., that Mr. Benjamin Moore, M.A., D.Sc., of Queen's University, Ireland, who is also a member of the staff of the Medical Research Council, is appointed first Professor of Bio-chemistry to the University. This appointment dates from October 1. Professor Moore was educated at Queen's College, Belfast, and Leipzig University. For five years he was on the staff of University College, and later went to Yale University as Professor of Physiology. More recently he has been Professor of Bio-chemistry to the University of Liverpool.

### THE BRITISH SCIENCE GUILD.

There was a large and distinguished gathering at the Goldsmith's Hall at the annual meeting of the British Science Guild on June 8, when addresses of considerable national importance were delivered by the Rt. Hon. Lord Sydenham, G.C.S.I., F.R.S., and the Rt. Hon. Lord Montagu of Beaulieu, who are respectively the retiring and the new Presidents of that body. An abstract of the first address is given on page 309.

### THE IMPERIAL WAR MUSEUM.

Never before has an exhibition of such absorbing interest been presented to the public as that opened by H.M. the King at the Crystal Palace last week. Every device for destruction, every means of defence is illustrated by a profusion of war trophies from sea, land, and air. Apart from the obvious interest of such a collection to the layman, there is a really marvellous display of medical exhibits, divided into military and naval sections. Not only does one gain a view, both comprehensive and invaluable for future professional work, of the many splendid services and inventions which were brought into action by our own great medical administrators, but the work—and indeed the ultimate straits—encountered by the enemy medical services are demonstrated in a manner that no literary description could ever hope to achieve. We are more than glad to learn that this war museum is to be a permanent one, and this being so it will serve a thousand educational purposes for years to come; but to-day, for those whose memories of war and its horrors are still fresh, we can recall no more enthralling afternoon's entertainment than a visit to the exhibition now open at Sydenham.

### NURSES AS ANÆSTHETISTS.

It is of interest to note, after the letters which a short time ago were appearing in the medical press on the subject of unqualified anæsthetists, that the American Association of Anæsthetists at a recent convention expressed itself as "unalterably opposed to the employment of lay anæsthetists, nurse anæsthetists, and all other types of anæsthetists who have not graduated for recognised medical or dental colleges, and have been licensed to practise medicine or dentistry." Although we had recently to protest against individual British institutions being repeatedly and unjustly cited during the process of bringing the danger of employing unqualified anæsthetists to the Government's notice, yet we are glad to see this American recommendation, and trust that the day is not far distant when we shall see this opinion generally supported by legal enactment.

### CERTIFIED MILK.

We understand that acting upon the principles we have recently described, milk bearing the description of "Grade A Certified Milk" is now being sold in Manchester in sealed 1-pint bottles at a cost of 7d. per pint—namely 3d. a pint more than the usual domestic price. This milk comes under Government guarantee from the farms directly and in the natural raw condition, without being either sterilised or pasteurised. The cattle concerned have all passed the tuberculin test and the houses and utensils have been certified according to the most exacting standards by the Ministry of Food. The date of sealing is stamped on every bottle. As a contemporary suggests, it would be of great interest if at certain welfare centres some of the poorer families could be assisted in obtaining this milk, and an attempt were thus made to estimate the extent to which this clean milk would prevent the incidence of summer diarrhœa, an illness the dire effect of which in connection with infant mortality is only too well known in this country.

### NEW RATES FOR PENSIONER PATIENTS.

On page 310 will be found the revised rates of payment for the in-patient treatment of war pensioners in voluntary hospitals. These have been formally sanctioned by the Treasury and have become operative as from January 1, 1920. It should be noted that the rates mentioned are the limits within which the Pensions Ministry will honour claims for payment to the hospitals, and individual hospitals are required to make their own separate arrangements with the Pensions Ministry through the Regional Commissioner of the district in which each hospital stands. It is suggested by the British Hospitals Association that during the continuance of the agreement a proportion not exceeding twenty per cent. of the gross receipts from the Ministry should be paid by each hospital to its Medical Staff Fund by arrangement with the medical staff, in order that the latter should be adequately remunerated for their work. The principle of adequate remuneration for this State work has already been decided on by the British Medical Association, and it is acknowledged by the Ministry of Pensions that the character of the work done for the pensioner is different from that of the charitable work hitherto performed by honorary medical staffs.

### BRITISH HOSPITALS' ASSOCIATION.

The annual meeting of the British Hospitals' Association has been fixed for Friday, June 25, at St. Thomas's Hospital at 3 P.M.

### SIR GILBERT BARLING'S PLAN.

The condition of the Hospital Sunday Fund in Birmingham is always interesting because the movement started there, and the city is supposed to be progressive. Denominational representatives met at the Council House to consider the Fund's contributions to hospitals. The Lord Mayor said that the Sunday Collection in 1919 was only £5,000, which was less than it had been twenty-five years ago, and still less than it was in 1878. Action must be taken. Sir Gilbert Barling said that fewer people went to church. He therefore proposed to increase the Sunday Fund collections by mapping the area covered by the movement into small sections, centring round some church, and that each place of worship should organise collections in its own district. To do this thoroughly Sir Gilbert said that each church should be able to enlist help from such voluntary workers as Red Cross detachments, Boy Scouts, and the St. John Ambulance Corps, whose duty it would be to canvass every householder. In this way those who never went to church would be reached, for a letter explaining the hospitals' needs would arrive at every house on the Saturday before Hospital Sunday. The plan was adopted unanimously. We hope that the circular letter will not only explain the hospitals' needs, but explain also the Sunday Fund movement, and the motive which inspires its work. It must not be allowed to degenerate into a mere collecting agency. We shall tell the story of the Sunday Fund in a Special Hospital Fund Number on June 26.

### SECTIONAL SUBSCRIPTIONS AT YORK.

To obviate the necessity of frequent appeals, which rarely produce more than a few thousand pounds, a sub-committee has been appointed to consider how to increase the income of the Yorkshire County Hospital. Its last farm was lately sold for £2,275, and the investment of this sum in trustee securities will bring the hospital a larger income. About £20,000 a year is now required, and the income at present is £14,000 only. The York Workpeople's Hospital Fund produced £840 during the past quarter, and this suggests that the organisation of sectional subscriptions should be taken seriously. The employers' subscription fund at Leeds, the newly-started Farmers' Fund at Hereford, the rural schoolchildren's weekly collection lately started in Suffolk, are instances which might be copied in York, where we should also like to see an organisation for the hospital's benefit among the retail shops. If the sub-committee will try less to invent new sources of income than to organise more methodically the old, it will have gone a long way to gain the extra £6,000 a year which are so badly needed.

### AN EMERGENCY GRANT FOR THE NATIONAL HOSPITAL.

As we pointed out last week, the National Hospital for the Paralysed and Epileptic is closed in part only. No more in-patients are to be admitted; but the country branch at East Finchley, the branches supported by the Ministry of Pensions, and the massage school and hostel continue open. In reply to a question from Sir C. Kinloch-Cooke last week in the House of Commons, Dr. Addison stated that King Edward's Hospital Fund was considering the National Hospital's application for an emergency grant. The application, which arrived apparently on the 10th inst., was, according to Dr. Addison, the first news received by the Fund of the intended closing. The Fund's decision will be anxiously awaited, for it will not be easy to make a substantial " emergency grant " without setting a precedent to which in the nature of things there must be a limit.

### NEW WELSH COTTAGE HOSPITAL.

For the benefit of the 4,000 workmen employed at the Ocean Collieries at Cwmparc, Mr. William Jenkins, a director of the company, is building a cottage hospital at Pentwyn, Treorchy. The hospital will have twenty beds, and work on the site has begun. The maintenance of the hospital is in part provided, since the men have agreed to a levy of a penny on the pound from their wages. The plans provide for future extension, and the whole scheme shows how much can be done by co-operation between all classes.

### PENSIONS FOR THE BLIND.

The Ministry of Health Bill now before Parliament provides for pensions for blind persons between the ages of fifty and seventy at the same rates and on the same conditions as attach to old age pensions, and like them graded in order of the means of the recipients. Ten shillings a week is given to a person whose income does not exceed £26 5s. 0d., and no pension is payable to the holder of an income of over £49 17s. 6d. It is officially estimated that the number of persons who will fulfil the statutory conditions is 8,400, and that the cost to be provided out of the Exchequer in a full year will not exceed £220,000. The Bill also gives power to local authorities to institute workshops, but these powers in the opinion of the National Institute for the Blind should not be permissive but obligatory. The institute, of which Sir Arthur Pearson is president, consider the proposed legislation, although inadequate in scope, is a step in the right direction.

### THE BRITISH HOSPITAL IN PARIS.

It is a sign of the times that an appeal has had to be issued on behalf of the Hertford British Hospital in Paris, which was founded and largely endowed by Sir Richard Wallace in 1879. The endowment of £3,878 per annum has become inadequate, and the hospital needs renovation. An additional sum of £2,000 a year is required, and the best means of raising this is through the British Colony in Paris, numbering 15,000, a committee of which manages the hospital. It is the only British hospital in Paris, and the appeal comes from the Embassy there. Hardly any institution is more closely connected with Franco-British life in Paris. The hospital grew out of the two sieges of Paris in 1870 and 1871 and since then has tended British sick. During the last war it was found very useful to the armies as it received military cases as well as civilians and when Sir John French's forces fell back to the Marne it was the Hertford Hospital which sheltered the many wounded who were sent to Paris. The Army cases received during the war numbered 1,500. The capital sum left to provide income for maintenance yields £3,878 per annum, and this sum has latterly proved inadequate for the needs of the hospital. Although the founder expressed the desire that in no way should the hospital have recourse to charity, it has been considered necessary to advise the public of the situation in the hope that the thousands who have benefited at its hands may be willing to come to its help.

### THE LAST OF THE WOUNDED.

Under the above title the *Evening Standard* of a few days ago had an article which reveals the fact that there are still between 2,000 and 3,000 wounded remaining in hospitals in London, at the Royal Herbert Hospital, Woolwich, at the 3rd and 4th London General Hospitals, at Queen Mary's Hospital, Roehampton, and elsewhere. The list given is, we know, not complete; but whatever the true numbers are, the men appear to have been almost completely forgotten by the public, and have in consequence but few of the attentions that were showered upon the wounded during the war. But there is a special department controlled by General Sir Richard Ewart at 27 Grosvenor Place, S.W. 1, the object of which is to provide the last of London's wounded with fresh air and amusement. Sir Richard appeals to people to lend their cars for one or two afternoons a week, to arrange picnics and garden parties, and generally to lend their aid to introduce a little happiness into the lives of the rear-guard of the vast army of wounded.

### "COLLECTORS" AND COTTAGE HOSPITALS.

There still exists, though the office is obsolescent, the paid collector, and one is to be appointed for the Whitstable Cottage Hospital. The chief supporter of the plan is Mr. F. Goldfinch, who said that the voluntary system of collecting subscriptions had failed, and added that he had had enough of voluntary collectors. There was, however, some disagreement, and this was strongest on the question of salary. A payment of £10 was suggested, plus five per cent. commission on the amount collected. A sub-committee is now to settle the matter, which suggests that, if the office lingers, it had not the weight of sound opinion behind it. The commission system is objectionable. A paid secretary is the proper plan.

### PRIORITY FOR PRISONERS OF WAR.

An interesting donation has been made to Mildenhall Cottage Hospital just in time to prevent a sale of stock to meet a small deficit. The donors are the Suffolk Prisoners of War Help Committee, who have sent £100 to be earmarked for Suffolk ex-Service men, with priority to those who have been made prisoners of war. To meet their wishes the Committee of the hospital has decided to admit all Suffolk patients who have served in the war, with priority to those who have been prisoners, without the customary fee.

### WHAT IT MEANS TO KEEP A COW.

Chelmsford Corporation Maternity and Child Welfare Committee has considered the acquisition of a herd of cattle so as to have its own milk supply. The medical officer of health reports, after inquiry, that if the object is to produce and place on the "market" a cheaper milk than is at present obtainable, dairy farming on a small scale may not succeed commercially. But if the object is to supply really tuberculosis-free milk of high quality for infants, the scheme might be considered favourably. In any event, the herd must be specially selected, and all tuberculous cattle eliminated by the tuberculin test. A specially designed cattle shed would be required. An experienced cowman, trained in cleanliness, would need to be engaged, and to ensure clean delivery a milkman and a milk store would probably be needed in addition. The medical officer consulted the veterinary inspector, who suggested the possibility of subsidising and branding a certain number of cattle in the possession of a local dairyman for exclusive use. These thorough precautions have led the Maternity Committee "to take no further action." The precautions, however, should set a useful standard for other committees.

### THIS WEEK'S DRUG MARKET.

The depressed state of business has become accentuated, and the downward movement is more pronounced. Japanese refined camphor and menthol are both cheaper again; since the fall begun a few months ago the prices of both have been reduced by one half. Potassium bromide is freely offered, but it is said that some of the samples are not up to standard; discrimination in buying is therefore necessary. Most of the synthetic drugs tend downwards, but the movement is slow; buyers, however, are holding off as far as possible. Lemon oil is rather cheaper, but the values of bergamot and orange oils are maintained. Cocaine is not in plentiful supply and the price is firm. Cod-liver oil is quiet and lower in price.

